# Effectiveness of COVID-19 Vaccines Against Hospitalization and Severe Disease in Children with Diabetes Mellitus During Pandemic and Post-Pandemic Eras

**DOI:** 10.3390/microorganisms14020501

**Published:** 2026-02-20

**Authors:** Laura G. Coelho, Lilian M. Diniz, Stella C. Galante, Cristiane S. Dias, Maria Christina L. Oliveira, Enrico A. Colosimo, Ana Cristina Simões e Silva, Fernanda N. Duelis, Maria Eduarda T. Bernardes, Daniela R. Martelli, Fabrício Emanuel S. Oliveira, Hercílio Martelli-Junior, Robert H. Mak, Eduardo A. Oliveira

**Affiliations:** 1 Health Sciences Postgraduate Program, Department of Pediatrics, School of Medicine, Federal University of Minas Gerais (UFMG), R. Engenheiro Amaro Lanari 389/501, Belo Horizonte 30310-580, MG, Brazil; coelholaura26@gmail.com (L.G.C.); lilianmodiniz@gmail.com (L.M.D.); stellacgalante@gmail.com (S.C.G.); profacristianedias@gmail.com (C.S.D.); chrismariana@gmail.com (M.C.L.O.); acssilva@hotmail.com (A.C.S.e.S.); duelisfernanda@gmail.com (F.N.D.); dudatbernardes@gmail.com (M.E.T.B.); 2 Department of Statistics, Federal University of Minas Gerais (UFMG), Belo Horizonte 31270-901, MG, Brazil; enricoc57@gmail.com; 3 Health Science/Primary Care Postgraduate Program, State University of Montes Claros (Unimontes), Montes Claros 39401-089, MG, Brazil; daniellareismartelli@yahoo.com.br (D.R.M.); fabricioemanuel1@hotmail.com (F.E.S.O.); hercilio.junior@unimontes.br (H.M.-J.); 4 Department of Pediatrics, Division of Pediatric Nephrology, University of California, San Diego, CA 92123, USA; romak@health.ucsd.edu

**Keywords:** COVID-19, diabetes mellitus, children, vaccine effectiveness, post-pandemic

## Abstract

Pediatric patients with SARS-CoV-2 infection are at an increased risk of severe disease and adverse outcomes. Nevertheless, comprehensive data on COVID-19 vaccine effectiveness (VE) in children with diabetes during the post-pandemic period remain limited. This study assessed the VE against severe COVID-19 outcomes during both the pandemic and post-pandemic phases in children with and without diabetes mellitus (DM). A cohort study based on population data was carried out, including all patients under 18 years of age with symptomatic SARS-CoV-2 infection as registered in the Brazilian national surveillance systems from February 2020 to June 2025. The main outcomes were hospitalization due to COVID-19 and severe illness, which included admission to the intensive care unit (ICU), need for invasive ventilation, and death. Utilizing a propensity score-matched cohort, we estimated the VE and the number needed to vaccinate (NNV) for a booster dose against these outcomes by comparing vaccinated and unvaccinated individuals, employing conditional logistic regression adjusted for confounding variables. The cohort comprised 3,730,007 pediatric patients with COVID-19, of whom 7675 (0.2%) had DM. At baseline, children with DM exhibited a significantly higher prevalence of hospitalization (11.2% vs. 2.0%), severe COVID-19 (6.4% vs. 0.6%), and mortality (1.9% vs. 0.1%) than those without DM (all *p* < 0.001). During the pandemic period, the adjusted VE was consistently higher in children with DM. Against severe disease, the VE was 72.8% (95% CI: 12.3–93.2) in the DM cohort compared with 45.7% (28.1–59.0) in the non-DM cohort. This increased effectiveness corresponded to a more favorable NNV; the NNV to prevent one severe case was 24 (95% CI: 12–232) for children with DM versus 243 (168–440) for those without DM. In the post-pandemic period, the VE remained significantly higher in the DM cohort. Against severe disease, the VE was 76.2% (11.5–93.5) for children with DM and 52.9% (32.7–67.1) for those without. The NNV to prevent one severe case was consistently lower in the DM cohort (8 vs. 591). In conclusion, a complete vaccination regimen, including a booster dose, substantially mitigated severe COVID-19 outcomes in children with DM in the pandemic and post-pandemic periods.

## 1. Introduction

During the COVID-19 pandemic, diabetes mellitus (DM) was established as a major risk factor for severe disease outcomes [1]. Research has established that SARS-CoV-2 infection markedly elevates the risk of severe illness, hospitalization, and mortality among adults with diabetes [2,3,4]. However, data on the outcomes of children with diabetes mellitus (DM) remain inconclusive. Generally, children with DM who contract SARS-CoV-2 tend to experience a mild course of COVID-19. For example, Nimri et al. [5] observed that young individuals with established type 1 diabetes (T1D) experienced mild COVID-19 infections, although factors such as elevated glucose levels during SARS-CoV-2 infection, advanced age, and comorbidities were associated with a prolonged disease course. Similarly, Demeterco-Berggren et al. [6] conducted a multicenter study involving patients with T1D and COVID-19 across 56 clinical centers in the United States. The study’s findings indicated that older patients were at an increased risk for severe COVID-19, whereas children and younger adults experienced milder illness, with no fatalities reported in these age groups. However, SARS-CoV-2 infection may still cause severe disease in those pediatric patients with DM and underlying conditions [7,8]. For example, we previously showed a higher prevalence of severe outcomes in children and adolescents with DM. Compared with children and adolescents without DM, pediatric patients with DM had a higher prevalence of ICU admission (46.6% vs. 26%), invasive ventilation (16.9% vs. 10.3%), and death (15% vs. 7.6%) [9]. Additionally, evidence suggests that COVID-19 may be associated with new-onset diabetes in children and may lead to a higher likelihood of developing diabetic ketoacidosis in children with DM [10,11].

COVID-19 vaccines effectively reduce severe outcomes in individuals with DM, albeit with potentially modestly reduced immunogenicity, particularly in individuals with poor glycemic control [12,13]. Consequently, major health authorities recommend COVID-19 vaccination, including updated boosters, for all individuals with DM [14,15]. Following the WHO’s declaration of the end of the public health emergency, SARS-CoV-2 has transitioned into an endemic state, which remains relatively understudied [16,17,18,19]. However, the virus continues to evolve and remains a relevant cause of morbidity and mortality due to acute respiratory infections [20,21]. For instance, we showed that children with a positive test for SARS-CoV-2 had a hazard of death three times higher than individuals with a negative test among children and adolescents hospitalized with severe acute respiratory infection from February 2020 to February 2023 in Brazil [22]. New variants continue to evolve to escape the neutralizing antibodies induced by both infection and vaccination, causing recurring, but generally less severe, waves of infection [23]. The highest burden of severe disease is now concentrated in specific high-risk groups: the elderly, immunocompromised individuals, and those with underlying chronic conditions including DM, cardiopulmonary disease, and obesity [24]. This ongoing evolution, coupled with an evolving immunological landscape, creates substantial uncertainty in the post-pandemic period. A key unresolved question is whether annual COVID-19 vaccine boosters continue to confer meaningful protection, underscoring the need for contemporary evidence on vaccine effectiveness (VE) to inform future public health policies [25].

Nevertheless, data on the effectiveness of COVID-19 boosters in children during the post-pandemic period are limited. Using a nationwide Brazilian cohort, we retrospectively assessed the real-world vaccine effectiveness in preventing severe COVID-19 outcomes among children with DM, comparing the pandemic and post-pandemic eras.

## 2. Materials and Methods

**Figure 1:**
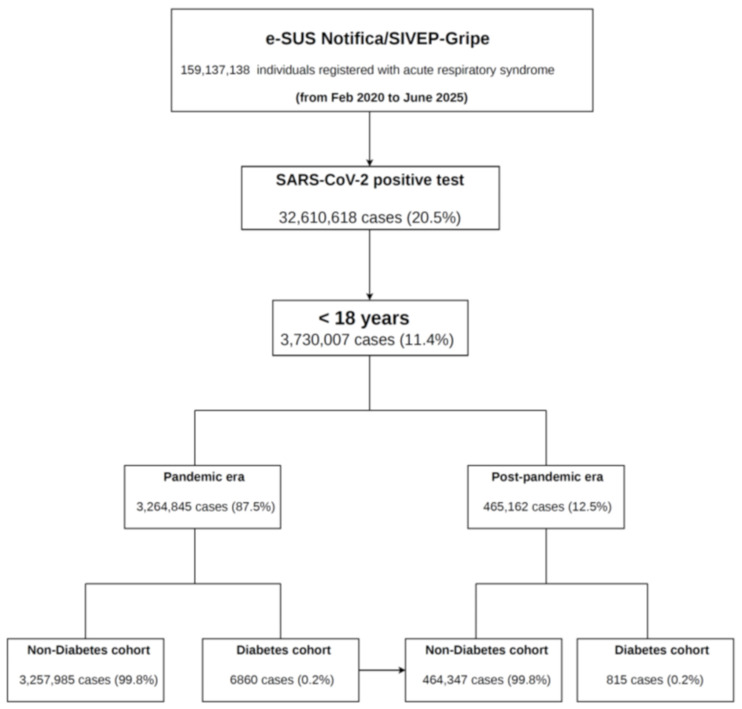
Flowchart of cohort selection.

*Study design, participants, and data sources*: We carried out a retrospective cohort study based on population data, utilizing information from two official Brazilian national COVID-19 surveillance systems supplied by the Ministry of Health: (1) e-SUS Notifica, which tracks non-hospitalized individuals, and (2) SIVEP-Gripe, which monitors those who are hospitalized. Our study included all individuals aged < 18 years with confirmed SARS-CoV-2 infection, as recorded in these systems from 20 February 2020 to 30 June 2025. SARS-CoV-2 infection was confirmed by a positive result from either quantitative reverse transcription PCR (RT-qPCR) or an antigen test. Comprehensive information regarding these systems is available at https://opendatasus.saude.gov.br/dataset (accessed on 30 June 2025). Complete information regarding database management and data handling is available elsewhere [26]. Figure 1 presents a flowchart illustrating the case-selection process used in our analysis.

*Exposure of interest*: The main exposure of interest was DM. We identified the existence of DM and other related chronic health conditions using specific database fields for comorbidities in the datasets.

*Covariates*: Demographic data, including age, sex, ethnicity, and geographic region, were collected. Age was stratified into three categories: 0–4, 5–11, and 12–17 years. Ethnicity was classified according to the Brazilian Institute of Geography and Statistics (IBGE) system into five groups: White, Black, Pardo, Asian, and Indigenous. The geographic regions were categorized into five official Brazilian macro-regions, each characterized by distinct historical, socioeconomic, and healthcare system attributes [27,28]. The clinical data gathered encompassed the date of COVID-19 symptom onset, date of hospitalization, signs and symptoms at baseline, and the presence of chronic health conditions.

Comorbidities were identified in dedicated fields in both datasets, which assigned the presence or absence of chronic medical conditions such as asthma, obesity, immunodeficiency, malignancies, and heart, lung, kidney, nervous system, and blood diseases. For our analysis, we classified comorbidity status as a binary variable (either present or absent) and by the number of conditions (none, one, or two or more conditions). Considering the nature of the national surveillance databases, we addressed missing data in the comorbidity fields by assuming their absence [27,29].

The study period was empirically divided into two phases: the pandemic era (1 February 2020, to 31 December 2022) and the post-pandemic era (1 January 2023, to 30 June 2025), corresponding to the decline in the number of reported cases and COVID-19-related deaths in Brazil. For each case, the index date for era assignment was the date of symptom onset. In instances where this information was unavailable, the date of the SARS-CoV-2 test was used as the index date.

*VaccinationStatus*: Vaccination status was defined as the receipt of vaccine doses at least 14 days before symptom onset, as this period is necessary to elicit an immune response. Individuals were classified into four categories: (1) unvaccinated, (2) one dose, (3) two doses, and (4) three or more doses. Patients with unknown vaccination status were excluded from the primary analysis. Information regarding the vaccination program in Brazil is detailed elsewhere [30].

*Outcomes:* The primary outcomes were (i) hospitalization due to SARS-CoV-2 infection and (ii) severe COVID-19, a composite endpoint defined as admission to the ICU, the need for invasive mechanical ventilation, or mortality. The effectiveness of the vaccines against hospital admissions and severe COVID-19 was estimated separately for two time periods (pandemic and post-pandemic eras) and was stratified by the presence of DM.

*Statistical analysis*: Continuous data are expressed as the median (IQR) or mean (SD) based on their distribution. Categorical data are presented as proportions. Group comparisons were performed using the chi-square test for proportions and the Mann–Whitney U test for non-normally distributed continuous variables.

The effectiveness of the vaccines in preventing hospitalization and severe COVID-19 cases was assessed using multivariable logistic regression models. These models were created for the entire cohort and for subgroups divided by the presence or absence of DM and by time periods, specifically during and after the pandemic. In these models, clinical outcomes were assigned as dependent variables. Vaccination status was the primary independent variable, and the models were adjusted for the following confounders: age, sex, ethnicity, geographic macro-region, predominant viral strain, and year of diagnosis/admission. Unvaccinated individuals were used as the reference group.

To complement this analysis and provide a robust estimate of the most complete vaccination schedule, propensity score matching (PSM) was performed, focusing on individuals who received three or more doses versus those who were not vaccinated. Four separate PSM analyses were conducted for the subgroups of interest (patients with and without DM during the pandemic and post-pandemic eras). For each PSM, the vaccination status (three or more doses versus unvaccinated) was designated as the exposure variable. The groups were matched for age, sex, ethnicity, geographic region, viral lineage predominance, and admission year using 1:1 nearest-neighbor matching without replacement and a caliper width of 0.2 standard deviations [31,32]. Following matching on the estimated propensity score, the balance between the vaccinated and unvaccinated groups was assessed using standardized mean differences (SMD) and graphical density plots of the propensity scores [33]. To perform a more robust analysis that adjusted for any residual imbalance, conditional logistic regression was conducted on the matched datasets. The outcome was the dependent variable, and the vaccination status was the primary predictor, with adjustment for potential residual confounding factors. After PSM, we obtained the average treatment effect (ATE) from the matched dataset and calculated the number needed to vaccinate (NNV) to prevent one outcome of interest [34]. For all analyses, VE was calculated as (1—adjusted odds ratio) × 100% and reported with 95% confidence intervals (CI). Data analysis was conducted using R software (version 4.3.0, The R Foundation), employing the MatchIt package for propensity score matching, while STATA (version 19) was used to calculate the average treatment effects. A *p*-value of less than 0.05 was considered statistically significant.

## 3. Results

### 3.1. Clinical Characteristics and Outcomes

The cohort comprised 3,730,007 pediatric patients with COVID-19, including 7675 (0.2%) with DM. Table 1 displays the demographic and clinical characteristics of the patients. Overall, the cohort was evenly distributed between the 5–11 (39.1%) and 12–17 (39.1%) age groups, with a smaller proportion (21.9%) aged 0–4 years. The majority of patients were male (54.5%), resided in the Southeast (40.0%) or South (25.0%) regions, identified as White (55.6%), and had no recorded comorbidities (96.3%). The most common presenting symptoms were cough (43.3%) and fever (40.8%).

### 3.2. Vaccine Effectiveness (Pandemic vs. Post-Pandemic Eras)

**Figure 2:**
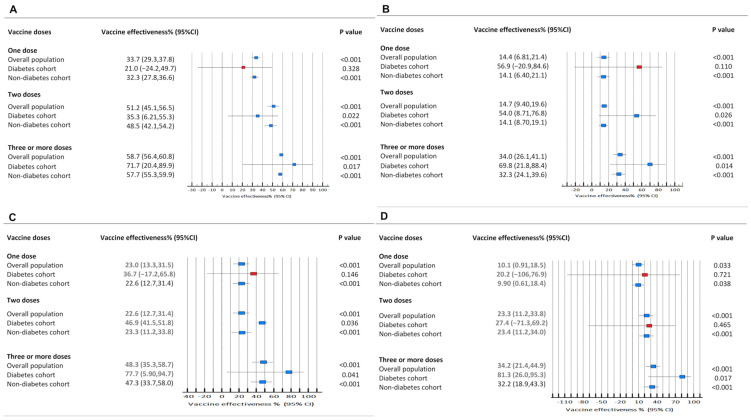
Estimated vaccine effectiveness against the outcomes of interest stratified by the pandemic and post-pandemic eras: hospitalization (Panel (**A**), pandemic; Panel (**B**), post-pandemic) and severe COVID-19 (Panel (**C**), pandemic; (**D**), post-pandemic). All models were adjusted for possible clinical and demographic confounders.

Compared with the non-DM cohort, the DM cohort had a higher proportion of females (57.6%) and a different regional distribution, with more patients from the Southeast region (49.0%) (*p* < 0.001). This group also had a significantly younger age distribution, with 46.8% in the 0–4 age group, and a slightly lower proportion of White ethnicity (49.2%). Dyspnea at baseline was markedly more prevalent in the DM cohort (20.5% vs. 6.3%, *p* < 0.001). All major comorbidities, including cardiovascular disease, obesity, and hypertension, were significantly more prevalent in patients with DM. Notably, the DM cohort exhibited a higher prevalence of full vaccination than did the non-DM cohort (Figure 2).

In general, individuals with DM exhibited a greater incidence of severe COVID-19 outcomes than those without DM, as evidenced by hospital admission rates (11.2% vs. 2.0%), severe COVID-19 cases (6.4% vs. 0.6%), and mortality (1.9% vs. 0.1%) (all *p* < 0.001).

We further evaluated the effectiveness of booster doses using propensity score matching (PSM). Overall, the PSM-adjusted estimates indicated a higher VE in children with diabetes. Post-matching, we compared vaccine effectiveness (VE) against specified outcomes and the number needed to vaccinate (NNV) during the pandemic and post-pandemic periods, stratified by DM status. As shown in Table 2, the estimated VE during the pandemic was consistently higher in children with DM. For instance, the VE against severe disease was 72.8% (95% CI, 12.3–93.2) in the DM cohort compared with 45.7% (95% CI, 28.1–59.0) in the non-DM cohort. This higher VE corresponded to a more favorable NNV; the NNV to prevent one severe case was 24 (95% CI, 12–322) for children with DM versus 243 (95% CI, 168–440) for those without.

In the post-pandemic era, the estimated VE remained statistically significant and higher in the DM cohort. For example, the VE against hospitalization was 89.0% (95% CI, 28.4–98.3) for children with DM and 60.6% (95% CI, 47.7–70.4) for those without. The NNV to prevent one hospitalization or severe COVID-19 was also consistently much lower in the DM cohort (Table 2).

## 4. Discussion

*Key points*: In this large-scale, population-based cohort study of 3.7 million pediatric patients in Brazil, including 7675 children with DM, we evaluated the effectiveness of COVID-19 booster doses in preventing hospital admissions and severe disease in this high-risk group. Using robust methods, binary logistic regression, and propensity score matching, we estimated vaccine effectiveness (VE) across different phases of SARS-CoV-2 dissemination (pandemic vs. endemic). As expected, children with DM were at a greater initial risk of experiencing severe outcomes than those without DM. Consequently, the VE was consistently higher in children with DM for both outcomes, irrespective of the viral phase or statistical method. Notably, in the propensity score-matched analysis, the highest effectiveness was observed in the post-pandemic period following booster doses (≥3 doses), with a VE of 89.0% (95% CI, 28.4–98.3) against hospital admission and 76.2% (95% CI, 11.5–93.5) against severe COVID-19. Our findings underscore that in the post-pandemic era, even within a changed immunological landscape, booster regimens maintained significant effectiveness in all pediatric patients, especially in children with DM. Finally, our results demonstrate that the NNV to prevent one severe outcome was highly favorable for children with DM, reinforcing the value of booster doses in this vulnerable population.

*Comparative analysis*: In contrast to the established elevated risk of severe COVID-19 in adults with DM, the risk in pediatric patients is debatable. Studies from high-income countries indicate that children with type 1 diabetes generally experience mild infections [5,6]. In contrast, our earlier research conducted in Brazil found that children with DM faced a greater risk of experiencing severe COVID-19 than those without diabetes. Confirming this, the current analysis, including a substantial cohort of hospitalized and non-hospitalized children, shows that patients with DM have a higher prevalence of severe disease and COVID-19-related mortality.

Regarding vaccination, it has been questioned whether individuals with DM can mount an effective immune response to COVID-19 vaccines, given that studies have suggested impaired cellular and humoral immunity in this population [35,36,37,38]. However, our findings highlight the substantial protective effect of full vaccination against hospitalization and severe outcomes in children with DM, which was sustained across both the pandemic and post-pandemic periods. The NNV to prevent one severe case was at least 34-fold lower in the DM cohort than in the non-DM cohort, indicating their higher initial risk and greater absolute benefit. When stratified by dose, the booster significantly increased VE, particularly in the post-pandemic period. This added benefit likely stems from the shorter interval between vaccination and exposure, which mitigates waning immunity, and from updated formulations targeting predominant variants.

Notably, vaccination significantly reduced the risk of severe disease in the DM cohort, with similar protection during the Delta (pandemic) and Omicron (post-pandemic) periods. While real-world data on VE in patients with DM post-pandemic are limited, our results align with those of a recent study by Sariol et al. [39], which found comparable immunogenicity after monovalent mRNA vaccination in children with and without DM. Although that study reported that boosters did not significantly increase neutralizing antibody titers against Omicron lineage variants, our real-world findings, consistent with other reports [40,41], confirm that primary vaccination provides effective protection against severe Omicron outcomes, with booster doses further enhancing VE.

*Limitations and Strengths*: This study was subject to several limitations. First, comorbidities in the official datasets were recorded only as binary variables (yes/no), lacking details on severity, control, or clinical history. This prevented the inclusion of critical features like DM phenotypes and glycemic parameters, potentially leading to an underestimation of VE [35,42]. Second, the generalizability of our findings may be limited, as the study was conducted in a middle-income country with a large vulnerable pediatric population [26], and VE is known to be context-dependent [43,44]. Third, vaccination dates for third or subsequent doses were unavailable. However, our sensitivity analysis revealed that misclassification predominantly affected individuals who received one or two doses, therefore exerting minimal influence on the VE estimates for those who received three or more doses. Fourth, the significant decrease in patients with DM during the post-pandemic period resulted in a small sample size, limiting our statistical power to analyze outcomes like mortality in this important subgroup. Finally, despite our diligent efforts, we were unable to identify other studies on the vaccination of children with diabetes in the post-pandemic period, which limited our ability to compare our findings with those of other populations.

Nevertheless, this study has some noteworthy features. Its overall large cohort size, including a substantial number of vaccinated children, enabled a comprehensive examination of outcomes and vaccine responses. Furthermore, this study was conducted among hospitalized and non-hospitalized patients with and without DM in Brazil during the pandemic and post-pandemic periods, providing valuable real-world evidence from a middle-income country.

*Policy implications*: Continuous review of the evidence is crucial for understanding the constantly changing epidemiological landscape and creating a solid foundation for medical guidelines and clinical decision-making. The findings of this study, based on a large population, can guide public health strategies and individual choices regarding COVID-19 vaccination in children with diabetes [45]. Specifically, we demonstrated that from a public health standpoint, the number of children who need to be vaccinated to prevent one severe COVID-19 outcome remains consistently low among children with DM. Considering the relatively low cost and established safety profile of the available vaccines, these findings support the consideration of annual booster schedules for high-risk groups, such as children with DM, as a highly efficient and cost-effective strategy.

## 5. Conclusions

During the pandemic phase, COVID booster vaccination was fundamental for preventing hospitalization and severe disease in all children. Importantly, this protective effect remained robust in all children in the post-pandemic context. During the pandemic period, the adjusted VE was consistently higher in children with DM. In the post-pandemic period, the VE remained significantly higher in the DM cohort. Furthermore, the NNV to prevent one hospitalization or severe case was consistently lower in children with DM, underscoring the efficiency and benefit of booster vaccination for this high-risk group. These findings provide strong evidence to support tailored booster vaccination strategies for children with DM.

## Figures and Tables

**Table 1:** Clinical and demographic features of children and adolescents with confirmed COVID-19 categorized by the presence of diabetes mellitus.

Covariates *	Overall (%) 3,730,007 (100.0)	Non-DM Cohort (%) 3,722,332 (99.8)	DM Cohort (%) 7675 (0.2)
Age (years)			
Median (IQR)	9.0 (5.0–14.0)	9.0 (5.0–14.0)	7.0 (1.0–14.0)
Mean (SD)	9.3 (5.2)	9.3 (5.2)	7.5 (6.5)
Age group (years)			
0–4	815,101 (21.9)	811,509 (21.8)	3592 (46.8)
5–11	1,458,324 (39.1)	1,457,094 (39.1)	1230 (16.0)
12–17	1,456,582 (39.1)	1,453,729 (39.1)	2853 (37.2)
Sex (n = 3,724,913)			
Male	2,031,465 (54.5)	2,028,219 (54.6)	3246 (42.4)
Female	1,693,448 (45.5)	1,689,033 (45.4)	4415 (57.6)
Region			
Southeast	1,491,076 (40.0)	1,487,314 (40.0)	3762 (49.0)
South	932,709 (25.0)	931,690 (25.0)	1019 (13.3)
Central-West	377,031 (10.1)	376,245 (10.1)	786 (10.2)
Northeast	636,856 (17.1)	635,268 (17.1)	1588 (20.7)
North	292,335 (7.8)	291,815 (7.8)	520 (6.8)
Ethnicity (n = 2,965,946)			
White	1,648,737 (55.6)	1,645,627 (55.6)	3110 (49.2)
Brown	1,177,553 (39.7)	1,174,788 (39.7)	2765 (43.7)
Black	77,658 (2.6)	77,340 (2.6)	318 (5.0)
Asian	61,288 (2.1)	61,156 (2.1)	132 (2.1)
Indigenous	710 (0.0)	709 (0.0)	1 (0.0)
Clinical presentation			
Fever	1,520,927 (40.8)	1,517,634 (40.8)	3293 (42.9)
Cough	1,616,742 (43.3)	1,612,356 (43.3)	4386 (57.1)
Dyspnea	236,071 (6.3)	234,494 (6.3)	1577 (20.5)
Odynophagia	1,035,656 (27.8)	1,032,888 (27.7)	2768 (36.1)
Number of Comorbidities			
None	3,593,339 (96.3)	3,593,339 (96.5)	0 (0.0)
1	129,500 (3.5)	123,816 (3.3)	5684 (74.1)
2	6266 (0.2)	4586 (0.1)	1680 (21.9)
3	902 (0.0)	591 (0.0)	311 (4.1)
Major comorbidities			
Pulmonary	58,735 (1.6)	58,355 (1.6)	380 (5.0)
Obesity	5956 (0.2)	5628 (0.2)	328 (4.3)
Cardiology	9405 (0.3)	8250 (0.2)	1155 (15.0)
Immunosuppression	6330 (0.2)	6185 (0.2)	145 (1.9)
Renal	1557 (0.0)	1520 (0.0)	37 (0.5)
SARS-CoV-2 strain			
Ancestral	609,332 (16.3)	607,708 (16.3)	1624 (21.2)
Gamma	1,087,021 (29.1)	1,084,529 (29.1)	2492 (32.5)
Delta	140,489 (3.8)	140,141 (3.8)	348 (4.5)
Omicron	1,893,165 (50.8)	1,889,954 (50.8)	3211 (41.8)
Admission Year #			
2020	590,014 (15.8)	588,449 (15.8)	1565 (20.4)
2021	1,252,910 (33.6)	1,250,003 (33.6)	2907 (37.9)
2022	1,421,921 (38.1)	1,419,533 (38.1)	2388 (31.1)
2023	329,655 (8.8)	329,107 (8.8)	548 (7.1)
2024	133,251 (3.6)	132,996 (3.6)	255 (3.3)
2025	2256 (0.1)	2244 (0.1)	12 (0.2)
Vaccine schedule (n = 3,437,430)			
None	2,759,411 (80.3)	2,754,055 (80.3)	5356 (74.6)
One	179,001 (5.2)	178,610 (5.2)	391 (5.4)
Two	421,044 (12.2)	419,914 (12.2)	1130 (15.7)
Three or more	77,974 (2.3)	77,670 (2.3)	304 (4.2)
Hospitalization			
No	3,654,309 (98.0)	3,647,492 (98.0)	6817 (88.8)
Yes	75,698 (2.0)	74,840 (2.0)	858 (11.2)
Severe COVID-19			
No	3,707,205 (99.4)	3,700,020 (99.4)	7185 (93.6)
Yes	22,802 (0.6)	22,312 (0.6)	490 (6.4)
Death (total)			
No	3,724,535 (99.9)	3,717,005 (99.9)	7530 (98.1)
Yes	5472 (0.1)	5327 (0.1)	145 (1.9)

* Data (n) in the first column represent the available data for those covariates with missing values (gender, ethnicity, and vaccine). All *p*-values < 0.001.

**Table 2:** Propensity score matching. Estimated vaccine effectiveness (VE) and number needed to vaccinate (NNV) to prevent one outcome in children with and without diabetes mellitus.

Period	Outcomes				
		Odds Ratio (95% CI)	VE (%) (95% CI)	ATE (95% CI)	NNV (95% CI)
Pandemic era Post-pandemic era	Hospitalization Diabetes cohort Non-Diabetes cohort Severe COVID-19 Diabetes cohort Non-Diabetes cohort Hospitalization Diabetes cohort Non-Diabetes cohort Severe COVID-19 Diabetes cohort Non-Diabetes cohort	0.309 (0.102–0.847) 0.756 (0.649–0.881) 0.272 (0.068–0.877) 0.543 (0.410–0.719) 0.110 (0.017–0.716) 0.394 (0.296–0.523) 0.238 (0.064–0.885) 0.471 (0.329–0.673)	69.1 (15.3–89.8) 24.4 (11.8–35.1) 72.8 (12.3–93.2) 45.7 (28.1–59.0) 89.0 (28.4–98.3) 60.6 (47.7–70.4) 76.2 (11.5–93.5) 52.9 (32.7.1)	−0.068904 (−0.118433, −0.019375) −0.0015551 (−0.003048, −0.000735) −0.0422418 −0.081378, −0.003105) −0.0041152 (−0.005952, −0.002272) −0.186813 (−0.289108, −0.084518) −0.0032506 (−0.004635, −0.001866) −0.131868 (−0.208792, −0.054944) −0.0016892 (−0.003117, −0.000261)	15 (8–52) 643 (328–1360) 24 (12–322) 243 (168–440) 5 (3–12) 307 (215–535) 8 (5–33) 591 (320–3800)

VE, vaccine effectiveness; ATE (average treatment effect), NNV (number needed to vaccinate). All *p*-values < 0.01.

## Data Availability

The original data presented in the study are openly available in https://opendatasus.saude.gov.br/dataset/ (accessed on 30 June 2025). Our analysis code is available upon request from the corresponding author (Eduardo A. Oliveira, eduolive812@gmail.com).

## Abbreviations

The following abbreviations are used in this manuscript:

COVID-19	Coronavirus disease 2019
DM	Diabetes mellitus
WHO	World Health Organization
SARS-CoV-2	Severe acute respiratory syndrome coronavirus 2
SIVEP-Gripe	Influenza Epidemiological Surveillance Information System
aOR	Adjusted odds ratio
CI	Confidence interval
NNV	Number Needed to Vaccinate
VE	Vaccine Effectiveness
ICU	Intensive Care Unit
ATE	Average treatment effect
SUS	Brazilian Public Health System
